# DLBCNet: A Deep Learning Network for Classifying Blood Cells

**DOI:** 10.3390/bdcc7020075

**Published:** 2023-04-14

**Authors:** Ziquan Zhu, Zeyu Ren, Siyuan Lu, Shuihua Wang, Yudong Zhang

**Affiliations:** 1 School of Computing and Mathematical Sciences, University of Leicester, Leicester LE1 7RH, UK; 2 Department of Information Systems, Faculty of Computing and Information Technology, King Abdulaziz University, Jeddah 21589, Saudi Arabia; 3 School of Computer Science and Technology, Henan Polytechnic University, Jiaozuo 454000, China

**Keywords:** blood cells, randomized neural network, ResNet50, generative adversarial networks

## Abstract

**Background:**

Blood is responsible for delivering nutrients to various organs, which store important health information about the human body. Therefore, the diagnosis of blood can indirectly help doctors judge a person’s physical state. Recently, researchers have applied deep learning (DL) to the automatic analysis of blood cells. However, there are still some deficiencies in these models.

**Methods:**

To cope with these issues, we propose a novel network for the multi-classification of blood cells, which is called DLBCNet. A new specifical model for blood cells (BCGAN) is designed to generate synthetic images. The pre-trained ResNet50 is implemented as the backbone model, which serves as the feature extractor. The extracted features are fed to the proposed ETRN to improve the multi-classification performance of blood cells.

**Results:**

The average accuracy, average sensitivity, average precision, average specificity, and average f1-score of the proposed model are 95.05%, 93.25%, 97.75%, 93.72%, and 95.38%, accordingly.

**Conclusions:**

The performance of the proposed model surpasses other state-of-the-art methods in reported classification results.

## 1 Introduction

The blood flowing in blood vessels is composed of blood cells and plasma. Blood is red because of red blood cells in the blood. Hemoglobin is a special protein that transports oxygen within red blood cells. It is a protein that makes the blood red and consists of globin and heme. Besides red blood cells, there are also white blood cells and platelets. Although they occupy a small share of blood, their functions are very important. These three kinds of blood cells account for 45% of the blood volume, and the remaining 55% of the volume is plasma.

Blood is distributed throughout the body and delivers nutrients to various organs. Naturally, it also stores important health information about the human body. The blood composition will change when there is a problem in our body. Therefore, the diagnosis of blood can indirectly help doctors judge a person’s physical state, which is the routine blood test we often hear of. The routine blood test mainly includes diagnosing red blood cells, white blood cells, and so on. Its significance is to find many early signs of systemic diseases, diagnose whether there is anemia or blood system disease, and reflect the hematopoietic function of bone marrow. Mainstream blood diagnosis is now used to detect white blood cell abnormalities. White blood cell analysis is an essential examination method for pathological blood samples and is an important indicator for detecting and observing diseases. White blood cell recognition is one of the important components of blood testing. By identifying the total number, relative ratio, and morphology of various white blood cells in the blood, we can determine whether there is a disease, the type of disease, and the severity of the disease. So, examining white blood cells is very important to understanding the body’s condition and diagnosing diseases.

With the unprecedented development of deep learning (DL), scholars have recently applied DL to the automatic analysis of blood cells. Over the past decade, DL methods have been put forward for diagnosing blood cells. Tran et al. [1] introduced a hybrid method to segment blood cells. The proposed method was created with pre-trained VGG-16. The end pooling layer of VGG-16 was replaced with semantic segmentation. The overall accuracy of the proposed method could achieve 89.45% accuracy. Habibzadeh et al. [2] put forward a computer-aided diagnosis (CAD) model to automatically classify blood cells. ResNet and Inception were used for feature extractions. Three technologies were proposed to pre-process images: color distortion, image flipping mirroring, and bounding box distortion. This system yielded 99.46% and 99.84% accuracy with ResNet 101 and ResNet V1 152. Tiwari et al. [3] built a novel model to classify blood cells automatically. There were two convolution layers, two pooling layers, and two fully connected layers. The self-built network achieved 78% accuracy for four categories of classification.

Alzubaidi et al. [4] proposed three self-made DL models to classify red blood cells. These three self-made models were composed of parallel and traditional convolution layers. There were some differences among these three models, such as different numbers of traditional and parallel convolution layers, different filters, and so on. The proposed models yielded 99.54% accuracy and 99.54% accuracy with SVM. Yildirim and Çinar [5] used four different four convolution neural networks (CNNs) with two filters to classify blood cells into four categories. Four CNNs were selected to extract features, which were ResNet50, DenseNet201, AlexNet, and GoogleNet. The median and Gaussian filters were used in this paper. DenseNet201 with a Gauss filter achieved 83.44% accuracy. Delgado-Ortet et al. [6] designed a new clinical decision support system to segment red blood cell images and detect malaria. This system included three steps: the segmentation, cropping, and masking of red blood cells and the classification of malaria. For the segmentation and classification, they designed two novel CNN models. One contained 7 fully convolutional layers, and another one was composed of 13 layers. The segmentation method obtained 93.72% accuracy, and the classification method achieved 87.04% specificity.

Jiang et al. [7] designed a DL model to detect blood cells based on the YOLO. They added the spatial and channel attention mechanisms in the YOLO and named this new network the attention-YOLO. The weighted feature vector replaced the original vector. Khouani et al. [8] proposed a DL model to classify blood cells. Firstly, they pre-processed the input to achieve better performance. Then, they tried five different convolution neural networks: Inception V3, VGG16, VGG19, ResNet50, and ResNet101. ResNet50 with the Adam optimizer could obtain the best performance. The proposed deep learning model obtained 95.73% precision and 0.9706 F-score. Patil et al. [9] introduced a hybrid deep learning model to classify white blood cells, which combined the canonical correlation analysis (CCA) and CNN-LSTM to achieve better performance. When Xception was selected as the backbone model, this system could achieve 95.89% accuracy.

H Mohamed et al. [10] put forward a combined model to classify white blood cells. Some pre-trained CNN models were implemented to extract features, and the traditional machine learning models were selected as the classifier. They tested ten pre-trained CNN models and six traditional machine-learning models. Finally, the MobileNet224 with logistic regression achieved 97.03% accuracy. Rahaman et al. [11] compared two models for detecting and counting blood cells, which were the YOLOv5m and YOLOv5s. Finally, the YOLOv5m and YOLOv5s achieved 0.799 precision and 0.797 precision. Sharma et al. [12] classified blood cells into four types based on DenseNet121. The normalization and data augmentation were implemented to improve the classification performance. This proposed model could achieve 98.84% accuracy, 98.85% sensitivity, and 99.61% specificity.

Aliyu et al. [13] introduced an effective model to classify red blood cells. Two phases were included in this model: firstly, the region of interest (ROI) in blood cells was identified, and secondly, AlexNet was selected for classification. The precision, specificity, sensitivity, and accuracy were 90%, 98.82%, 77%, and 95.92%, respectively. Kassim et al. [14] designed a hybrid pipeline to detect red blood cells. U-Net and Faster R-CNN were the vital parts of this hybrid pipeline. The detection accuracy by the proposed model was 97%. Muthumanjula and Bhoopalan [15] built a novel DL network to detect white blood cells. Firstly, the CMYK-moment approach was implemented to identify ROI. Then, CNN was utilized to achieve features. This novel deep learning network yielded 96.41% accuracy.

Shahin et al. [16] put forward a new method (WBCsNet) to identify white blood cells. Several CNN models were utilized to extract features. The SVM was used as the classifier. The proposed WBCsNet achieved 96.1% accuracy. Ekiz et al. [17] selected two models to detect white blood cells. First, CNNs were applied to extract features. Second, the extracted features were used as the input to SVM for classification. The Con-SVM model could achieve 85.96% accuracy. Ammar et al. [18] applied seven different combinations of CNN models with other traditional classifiers to classify blood cells, including KNN, SVM, and AdaboostM1. Finally, the CNN-AdaboostM1 yielded 88% accuracy.

Singh et al. [19] designed a self-made CNN model which included two convolutional layers, two pooling layers, and two fully connected layers. They tested this self-made CNN with different epochs. When the epoch was chosen as 100, this self-made CNN could obtain 86% accuracy. Liang et al. [20] combined CNN models with other networks for the multi-classification of white blood cells. The pre-trained CNN models were chosen to be the feature extractors. Then, recurrent neural networks were implemented as the classifiers. In the experiments, the Xception-LSTM could achieve 90.79% accuracy.

From the above analysis, a sea of DL models could yield certain blood cell diagnosis performances [21–23]. However, there are still some deficiencies in these models. Some of them would use handcrafted features [24–27], but these features could not be the ideal maps for blood cell diagnosis. Meanwhile, DL models could take a lot of time to complete the experiments because of the massive layers and parameters. Furthermore, the over-fitting problem is another major concern when these DL models are trained on medical image datasets, which only contain a small number of images. This paper demonstrates a novel DL model (DLBCNet) for the multi-classification of blood cells. We use pre-trained ResNet50 as the backbone to extract ideal features. There are two ways to deal with the overfitting problem in this paper. First, we propose a new model (BCGAN) to generate synthetic images to create a larger dataset. Second, the proposed ETRN not only has a simpler structure but also achieves better performance than common DL models. The main contributions of our work are given as follows:

- The pre-trained ResNet50 is implemented to extract ideal features by comparing it with other CNN models;
- The proposed BCGAN is used to generate synthetic images to alleviate the overfitting problem;
- We propose ETRN to enhance the robustness with the ensemble strategy of combining three individual networks;
- We propose a novel DL model to classify blood cells, which is named DLBCNet.

The structure of this paper is presented as follows. Section 2 talks about the materials. The methodology is shown in Section 3. The experiment and results are given in Section 4. Section 5 concludes this paper.

## 2 Materials

**Figure 1:**
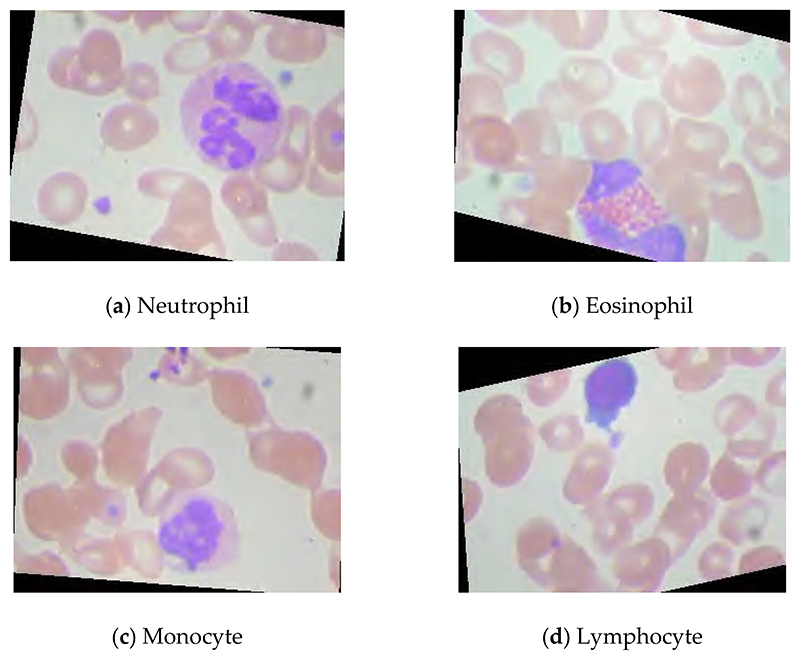
The images of these four classes of blood cells.

The dataset is available on this website (https://www.kaggle.com/datasets/paultimothymooney/blood-cells (accessed on 2 January 2023)). This public blood cell dataset comprises 12,500 augmented images of blood cells. There are four different blood cell classes: neutrophil, eosinophil, monocyte, and lymphocyte. Each blood cell class can have approximately 3000 images. The images of these four classes of blood cells are presented in Figure 1.

Neutrophils are white blood cells with the highest proportion in peripheral blood, accounting for more than half of white blood cells [28]. They are important components of innate immunity and important effector cells of immune defense. Eosinophils are a kind of white blood cell. Although the number of eosinophils in the blood accounts for less than 5%, they greatly kill bacteria and parasites [29]. Monocytes account for about 3%~8% of the number of white blood cells. They are the largest blood cells in the blood and an important part of the body’s defense system [30]. Lymphocyte is a kind of white blood cell, which is the smallest white blood cell [31]. It is an important cellular component of the immune response function of the body and the main executor of almost all immune functions of the lymphatic system.

## 3 Methodology

### 3.1 Feature Learning

Table 1 enumerates the acronyms and provides full explanations. The DL models have achieved remarkable success in various fields, such as natural language processing (NLP), image segmentation, etc. Modern, powerful computing capability makes it possible to have deeper DL networks. These deeper networks often lead to better performance. In recent decades, many epoch-making CNNs have been designed, such as AlexNet [32], ResNet [33], MobileNet [34], and so on.

For image recognition, feature extraction is an important process. Because the volumes of the images are usually too large with excessive information, it is difficult to extract the discrimination rate features. The distribution of features in latent space directly determines the complexity of image classification. With the continuous progress of computer science, CNN models have been the leading solution to the problem of image feature extraction.

**Figure 2:**
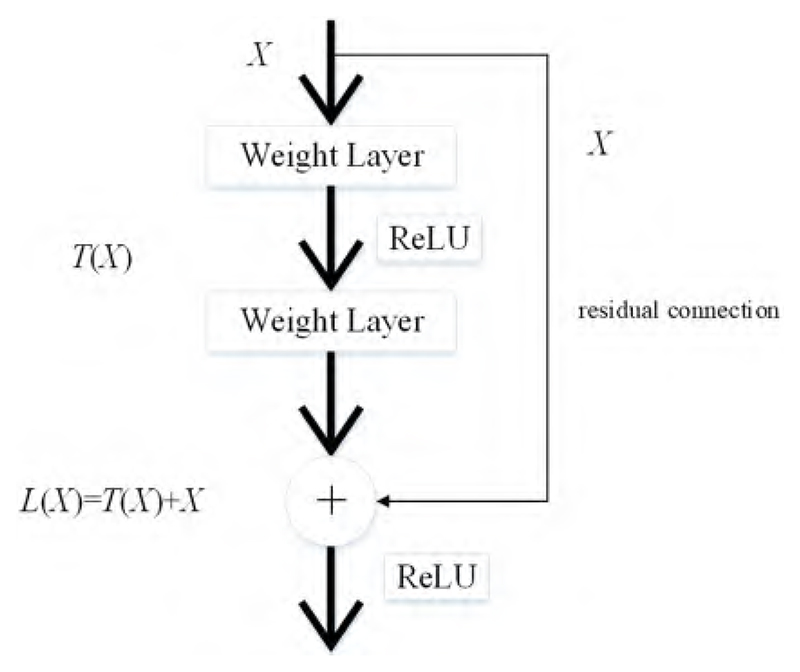
The structure of the residual connection.

It is time-consuming to train CNN models from scratch. Therefore, transfer learning is a feasible method for extracting image features. These pre-trained CNN models are transferred for feature extraction of cell images because they have strong image representation learning ability. ResNet50 is implemented as the backbone model in this paper. The residual connection in ResNet50 is one of the most important inventions in the recent decade of computer science, and can directly connect two non-adjacent layers to complete identity mapping. The framework of the residual connection is given in Figure 2.

Given *X* as the feature maps from the previous layer, the learned feature is set as *L*(*X*). *T*(*X*) is obtained through the residual connection as follows:

(1) T(X)=L(X)−X.

The learned feature with the conversion of the above formula is expressed as follows:

(2) L(X)=L(X)+X.

**Figure 3:**
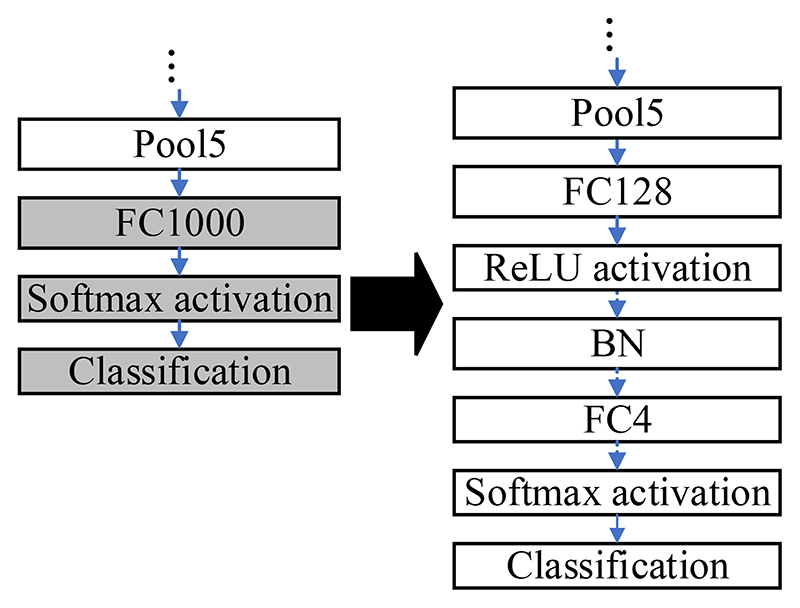
The modifications to the pre-trained ResNet50.

The ResNet50 pre-trained on the ImageNet dataset is modified due to differences in the dataset. The pre-trained ResNet50 is applicable to distinguish 1000 categories of images. Nevertheless, the public blood cell dataset in this paper has only four categories in total: neutrophil, eosinophil, monocyte, and lymphocyte. The modifications to the pre-trained ResNet50 are presented in Figure 3.

The last three layers of the pre-trained ResNet50 are removed, and six layers are added, which are ‘FC128’, ‘ReLU’, ‘BN’, ‘FC4’, ‘Softmax’, and ‘Classification’. The parameters in the pre-trained ResNet are frozen except those in the last three layers. Some buffer layers, which are ‘FC128’, ‘ReLU’, and ‘BN’, are inserted between ‘Pool5’ and ‘FC4’ because there are 1000 and 4 output nodes in the ImageNet dataset and the blood cell dataset, accordingly. The buffer layers can smooth the reduction procedures of the dimensions. The modified ResNet50 is fine-tuned by the blood cells dataset.

### 3.2 Proposed BCGAN

CNN models proved promising when implemented in image recognition and yielded excellent results in big datasets, such as ImageNet [35], CoPhIR [36], and so on. However, the overfitting problem [37] is often encountered when CNN models are applied to small image datasets. The samples of medical datasets are rarely compared with some datasets, such as the ImageNet dataset. It is very time-consuming to create labeled medical datasets.

**Figure 4:**
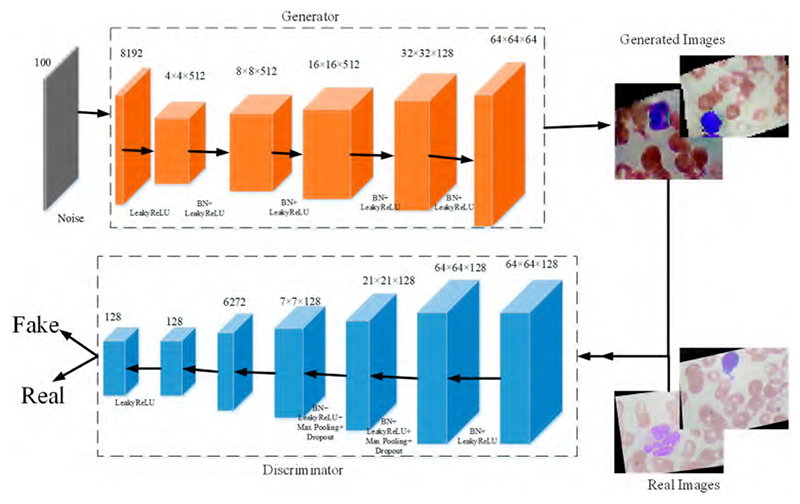
The proposed BCGAN.

When researchers employ supervised machine learning models in medical image recognition, the limited labeled dataset can especially restrain the performance. Meanwhile, many studies [38–41] prove that CNN models can achieve better performance with more data. To deal with these problems, we propose a new generative adversarial network for blood cells (BCGAN) to cope with the limited dataset issue, as shown in Figure 4.

The proposed BCGAN is inspired by generative adversarial networks (GANs) [42]. Two components form the proposed BCGAN, which are the generator *G* and discriminator *D*. The generator *G* obtains the random noise and generates synthetic images. The discriminator *D* is used to identify whether the image is real or fake. The generator *G* and discriminator *D* compete with each other. Generator *G* generates synthetic images similar to the real image as much as possible so that discriminator *D* cannot distinguish the generated images as fake. Discriminator *D* tries to improve the accuracy of identifying the real images and the generated images as much as possible. The proposed BCGAN generates synthetic blood cell images when the discriminator is unable to find the differences between generated images and real images.

Given the data *x*, *p_data_* is denoted as the probability distribution, and the noise is presented as *p_z_*(*z*). The loss function *F*(*D, G*) is calculated as follows:

(3) $$\min_{G}\max_{D}F(D,G)=E_{x∼p_{\text{data}\;}(x)}[\log D(x)]+E_{z∼p_{z}(z)}[\log\;(1-D(G(z)))],$$

where the discriminator *D* tries to maximize *D*(*x*) from generated data *x* ∼ *p_data_*(*x*), and the generator *G* is trained to maximize *D*(*G*(*z*)). During the training of the BCGAN, the generator *G* improves its ability to generate more realistic images, and the discriminator *D* enhances the ability to differentiate the real images and generated images. Therefore, the entire training process of BCGAN can be considered as a minimax game between the generator *G* and the discriminator *D*.

In the proposed BCGAN, the convolutional layers are used to extract features. The LeakyReLu is implemented to add nonlinearity. Max pooling is a common strategy to downsample the extracted features. Batch normalization (BN) is chosen to alleviate the gradient disappearance. The overfitting problem can be alleviated by adding the dropout. The BCGAN is specially designed for blood cell images. The pseudocode of the proposed BCGAN is introduced in Algorithm 1. The main contributions of the BCGAN are as follows:

- Five filters are added to increase the ability of the generator to capture more high-level features;
- Additional dropout layers can be helpful in avoiding the overfitting problem;
- The checkboard patterns can be alleviated by the larger kernel size;
- Batch normalization (BN) is inserted into the generator and discriminator to deal with the overfitting problem.

Algorithm 1 **Generative adversarial network for blood cell (BCGAN).**

**Input**:

      The *n* noise samples (*z*) from *p_z_*,

      *n* real samples (*x*) from *p_data_*,

      number of steps (*S*),

      the number of training iterations (*T*).

**Output**:

      Synthetic images

**for** *t* = 1, …, *T* **do**

      **for** *s* = 1, …, *S* **do**

            1.    Sample mini-batch of noise samples (*z*) from *p_z_*.

            2.    Sample mini-batch of real samples (*x*) from *p_data_*.

            3.    Update the discriminator D by:

$$\frac{1}{n}\sum_{i}^{n}[\log D(x_{i})]+[\log\;(1-D(G(z_{i})))].$$

      **end**

      4.    Update the generator G by:

$$\frac{1}{n}\sum_{i}^{n}\log\;(1-D(G(z_{i})))$$

**end**

We use BCGAN to generate 3000 synthetic images for each class. These synthetic images are mixed with original images to create a new dataset (named mixed-BCGAN dataset). At the same time, we use GANs [42] to generate 3000 synthetic images for each class, which are combined with original images to produce the mixed-GAN dataset.

The comparison of these three datasets is shown in Table 2. The training sets of the mixed-GAN and mixed-BCGAN datasets contain 3000 synthetic images and about 2175 original images for each class. The testing sets of the mixed-GAN and mixed-BCGAN datasets are composed of 933 original images per class. The original dataset’s training set and testing set cover about 2178 and 933 original images per class, respectively.

### 3.3 Proposed ETRN

For the classification of blood cells, three randomized neural networks (RNNs) are implemented to replace the last five layers of the backbone model: extreme learning machine (ELM) [43], random vector functional link (RVFL) [44], and Schmidt neural network (SNN) [45]. These three RNNs merely include three layers: the input layer, hidden layer, and output layer. The training of RNNs can be faster than traditional CNN models benefiting from the simple structure. Compared with the back-propagation neural network, because the weights and bias in RNNs were randomly initialized and fixed in training and the outputs can be calculated by pseudo-inverse, it is unnecessary to update the parameters based on back-propagation, which can shorten the training time. On the other hand, these three RNNs used to replace the last five layers can improve the classification performance.

**Figure 5:**
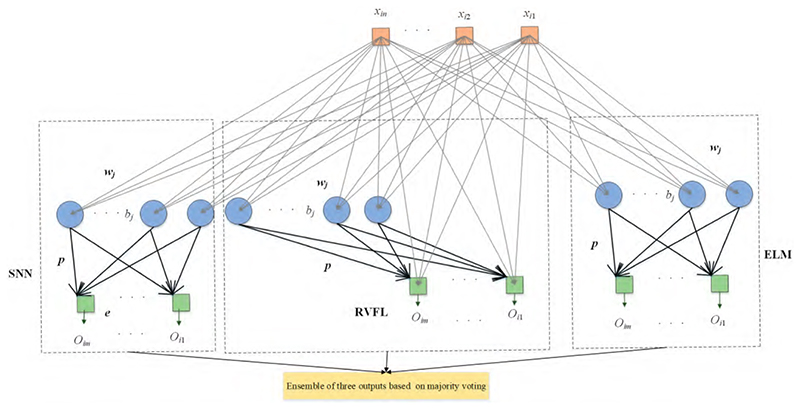
The structure of ETRN.

Ensembles of neural networks are usually recognized to be more robust and accurate compared with individual networks, even though these individual networks can obtain promising results. RNNs are regarded as unstable networks whose performance greatly varies with small perturbations because of the randomized weights and bias. In this situation, we propose a novel network named ETRN to improve classification performance. The structure of the proposed ETRN is given in Figure 5. The pseudocode of the proposed ETRN is shown in Algorithm 2. In the ETRN, three RNNs are trained and then combined with majority voting.

The strategy of the ensemble of three RNNs based on majority voting is given below:

(4) $$L(c)=\{\begin{array}{c} {\text{R}}_{a},\;\text{if}\;\exists {\text{R}}_{a}=={\text{R}}_{b},a,b\in \{e,v,s\}\\ {\text{R}}_{e},\;\text{otherwise}\; \end{array},$$

where *c* is the image in the dataset, *L*(*c*) is represented as the ensemble output, and ***R**_e_*, ***R**_v_*, and ***R**_s_* are denoted as the predictions from ELM, RVFL, and SNN, accordingly.

Algorithm 2 **The pseudocode of the proposed ETRN.**

**Input**:

      A training set (***x_i_***, ***y_i_***),

      activation function *g*(),

      hidden node number *Z*

**Output**:

      Predictions of images

**Individual network training**

      **ELM training**

            1.  Randomly generate parameters (***w_j_***, *b_j_*).

            2.  Calculate **H_ELM(*i*)_**:

$$H_{\text{ELM}(i)}=\sum_{j=1}^{Z}g(w_{j}x_{i}+b_{j}),i=1,\ldots ,N.$$

            3. Determine **P_ELM_**:

$$p_{\text{ELM}\;}=H_{\text{ELM}}^{+}Y.$$

            4.  Calculate the predictions of images of ELM.

      **end**

      **RVFL training**

            1. Randomly generate parameters (***w_j_***, *b_j_*).

            2.  Calculate **H_RVFL(*i*)_:**

$$H_{\text{RVFL}(i)}=\sum_{j=1}^{Z}g(w_{j}x_{i}+b_{j}),i=1,\ldots ,N.$$

            3.  Contact the input and the output of the hidden layer:

$$M_{\text{RVFL}(i)}=\text{concat}(X,H)\text{.}\;$$

            4.  Determine **P_RVFL_**:

$$p_{\text{RVFL}\;}=H_{\text{RVFL}}^{+}Y.$$

            5.  Calculate the predictions of images of RVFL.

      **end**

      **SNN training**

            1.  Randomly generate parameters (***w_j_***, *b_j_*).

            2.  Calculate **H_SNN(*i*)_**:

$$H_{\text{SNN}(i)}=\sum_{j=1}^{Z}g(w_{j}x_{i}+b_{j}),i=1,\ldots ,N.$$

            3. Determine (**P_SNN_**, ***e*_SNN_**):

$$(p_{\text{SNN}},e_{\text{SNN}})=H_{\text{SNN}}^{+}Y.$$

            4.  Calculate the predictions of images of SNN.

      **end**

**end**

**Ensemble training**

      Calculate the results based on three RNNs:

$$L(c)=\{\begin{array}{c} {\text{R}}_{a},\;\text{if}\;\exists {\text{R}}_{a}=={\text{R}}_{b},a,b\in \{e,v,s\}\\ {\text{R}}_{e},\;\text{otherwise}\; \end{array}$$

**end**

The calculations of ELM can be summarized in three steps. Given *N* samples with *i*-th samples as (***x_i_***, ***y_i_***):

(5) $$x_{i}=(x_{i1},\ldots ,x_{in}{)}^{\text{T}}\in {\text{R}}^{n},i=1,\ldots ,N,$$

(6) $$y_{i}=(O_{i1},\ldots ,O_{im}{)}^{\text{T}}\in {\text{R}}^{m},i=1,\ldots ,N,$$

The randomized weights and bias are fixed during the training process, and the outputs of the hidden layer are computed below:

(7) $$H_{\text{ELM}(i)}=\sum_{j=1}^{Z}g(w_{j}x_{i}+b_{j}),i=1,\ldots ,N.$$

where ***w_j_*** is the weight between the input and the *j*-th hidden node, *b_j_* is the bias of the *j*-th hidden node, *g*() is the activation function, and *Z* is denoted as the number of hidden nodes.

The output weight is calculated as follows:

(8) $$p_{\text{ELM}}=H_{\text{ELM}}^{+}Y\text{.}\;$$

where $H_{\text{ELM}}^{+}$ is the pseudo-inverse matrix of **H_ELM_** and **Y** = (***y*_1_**, … ***y_N_***)^T^ is the ground-truth label matrix of the dataset.

The structure of RVFL has direct connections between the input and output, as shown in Figure 5. Even though the structure is different, the calculation steps are the same. First, calculate the hidden layer output as follows:

(9) $$H_{\text{RVFL}(i)}=\sum_{j-1}^{Z}g(w_{j}x_{i}+b_{j}),i=1,\ldots ,N.$$

The input of the output layer is different because there are direct connections as follows:

(10) $$M_{\text{RVFL}(i)}=\text{concat}(X,H)\text{.}\;$$

where **X** = (***x***_1_, … ***x_N_***)^T^ is the input matrix.

The output weight of RVFL is calculated as follows:

(11) $$P_{\text{RVFL}}=H_{\text{RVFL}}^{+}Y.$$

The structure of SNN is similar to ELM. The only difference between these two RNNs is that there is an output bias in the SNN. The framework of SNN is presented in Figure 5.

The output of the hidden layer in SNN can be computed as follows:

(12) $$H_{\text{SNN}(i)}=\sum_{j=1}^{Z}g(w_{j}x_{i}+b_{j}),i=1,\ldots ,N.$$

The equation for SNN with output bias is shown below:

(13) $$(p_{\text{SNN}},e_{\text{SNN}})=H_{\text{SNN}}^{+}Y.$$

### 3.4 Proposed DLBCNet

We propose a novel DL network to diagnose blood cells (DLBCNet). Collecting a large number of labeled blood cell images to train DL modes is a challenge due to cost and time restrictions. We propose a new specifical model for blood cells (BCGAN) to cope with this challenge. More filters and dropout layers for each layer are added to capture more high-level features. Additional dropout layers and BN are added to avoid the overfitting problem.

Meanwhile, the checkboard patterns can be alleviated by the biggest kernel size. The ResNet50 pre-trained on the ImageNet dataset is implemented as the backbone model in this paper, which is modified and fine-tuned based on blood cells because of the difference between the ImageNet dataset with the blood cell dataset used in this paper. The modified ResNet50 is applied as the feature extractor. The last five layers of the modified ResNet50 are substituted with three RNNs (ELM, RVFL, and SNN). These three RNNs are used for classification. The sample structure and randomized weights of RNNs can reduce training time.

**Figure 6:**
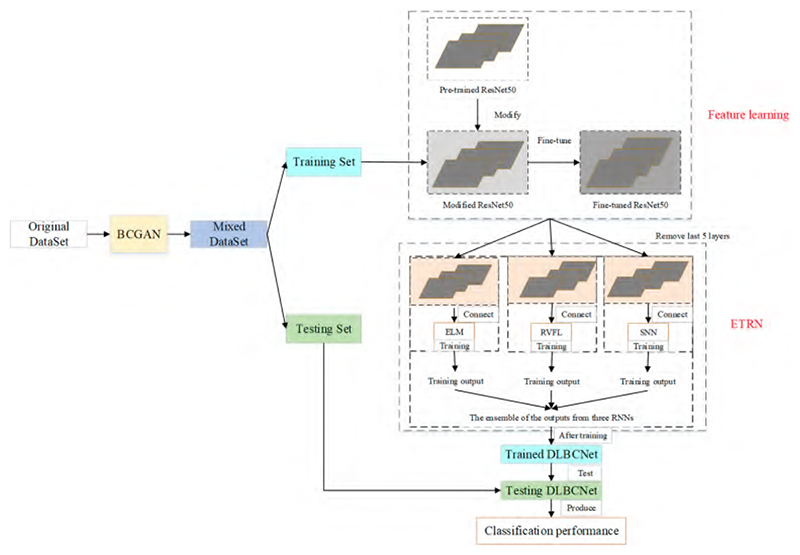
The overview of the proposed DLBCNet.

Nevertheless, the RNN is considered an unstable neural network due to some randomized operations. We propose the ETRN by combining three RNNs based on the majority voting to improve the robustness and the generalization performance. The overview of the proposed DLBCNet is demonstrated in Figure 6. The pseudocode of the DLBCNet is illustrated in Algorithm 3.

Algorithm 3 **The pseudocode of the DLBCNet.**

Step 1: Propose BCGAN.

      Step 1.1 Generate 3000 synthetic images per class based on the original dataset.
      Step 1.2 Mix synthetic images with original images to create the mixed dataset.
      Step 1.3 Divide the mixed dataset into training and testing sets.

Step 2: Generate the modified ResNet50.

      Step 2.1 Load pre-trained ResNet50.
      Step 2.2 Remove FC1000, softmax, and classification layer from the pre-trained ResNet50.
      Step 2.3 Add FC128, ReLU, BN, FC4, softmax, and classification layer.

Step 3: Fine-tune the modified ResNet50.

      Step 3.1: Input is the training set.
      Step 3.2: Target is the corresponding label.

Step 4: Replace the last five layers of the fine-tuned ResNet50 with three RNNs.

Step 5: Propose ETRN.

      Step 5.1: Ensemble the predictions of the three RNNs
      Step 5.2: Majority voting of the ensemble of the predictions from the three RNNs.
      Step 5.3: The whole network is named DLBCNet.

Step 6: Test the trained DLBCNet on the testing set.

Step 7: Report the classification performance of the trained DLBCNet.

### 3.5 Evaluation

Five multi-classification measurements are applied to evaluate the proposed DLBCNet, which are average accuracy, average sensitivity, average precision, average specificity, and average f1-score for four classes. First, the formulas of accuracy, sensitivity, precision, specificity, and f1-score per class are defined as follows:

(14) $$\left\{ \begin{array}{l} \begin{array}{l} \text{accuracy}(\partial )=\frac{\text{TP}(\partial )+\text{TN}(\partial )}{\text{TP}(\partial )+\text{FP}(\partial )+\text{TN}(\partial )+\text{FN}(\partial )}\\ \;\text{precision}\;(\partial )=\frac{\text{TP}(\partial )}{\text{TP}(\partial )+\text{FP}(\partial )} \end{array}\\ \begin{array}{l} \text{specificity}(\partial )=\frac{\text{TN}(\partial )}{\text{TN}(\partial )+\text{FP}(\partial )}\\ \;\text{sensitivity}\;(\partial )=\frac{\text{TP}(\partial )}{\text{TP}(\partial )+\text{FN}(\partial )} \end{array}\;,\partial =1,\ldots ,4,\\ \text{f}1-\text{score}(\partial )=\frac{2\times \;\text{precision}\;(\partial )\times \text{sensitivity}(\partial )}{\;\text{precision}\;(\partial )+\;\text{sensitivity}\;(\partial )} \end{array}\right.$$

where *∂* is denoted as the number of classes in this paper. For multi-classification, one class is defined as the positive class. The other three classes are negative classes. The average accuracy, average sensitivity, average precision, average specificity, and average f1-score are calculated below:

(15) $$\left\{ \begin{array}{l} \begin{array}{l} \;\text{average-accuracy}\;=\frac{{\text{Σ}}_{\partial =1}^{4}\text{accuracy}(\partial )}{4}\\ \;\text{average-precision}\;=\frac{{\text{Σ}}_{\partial =1}^{4}\;\text{precision}\;(\partial )}{4} \end{array}\\ \begin{array}{l} \;\text{average-specificity}\;=\frac{{\text{Σ}}_{\partial =1}\;\text{specitcity}\;(\partial )}{4}\;,\partial =1,\ldots ,4.\\ \;\text{average-sensitivity}\;=\frac{\sum_{\partial =1}^{4}\;\text{sensitivity}\;(\partial )}{4} \end{array}\\ \;\text{average-f1-score}\;=\frac{\sum_{\partial =1}^{4}\text{f}1-\;\text{score}\;(\partial )}{4} \end{array}\right.$$

The receiver operating characteristic (ROC) curve and the area under the curve (AUC) are used in this paper to evaluate the proposed model.

## 4 Experiment Settings and Results

### 4.1 Experiment Settings

The hyper-parameter setting of the proposed DLBCNet is presented in Table 3. The max-epoch is set to 1 to avoid the overfitting problem. The mini-batch size is ten because of the memory size of our device. The initial learning rate is 10^−4^ based on experience. The hidden nodes in the hidden layer are set as 400.

### 4.2 The Performance of DLBCNet

**Figure 7:**
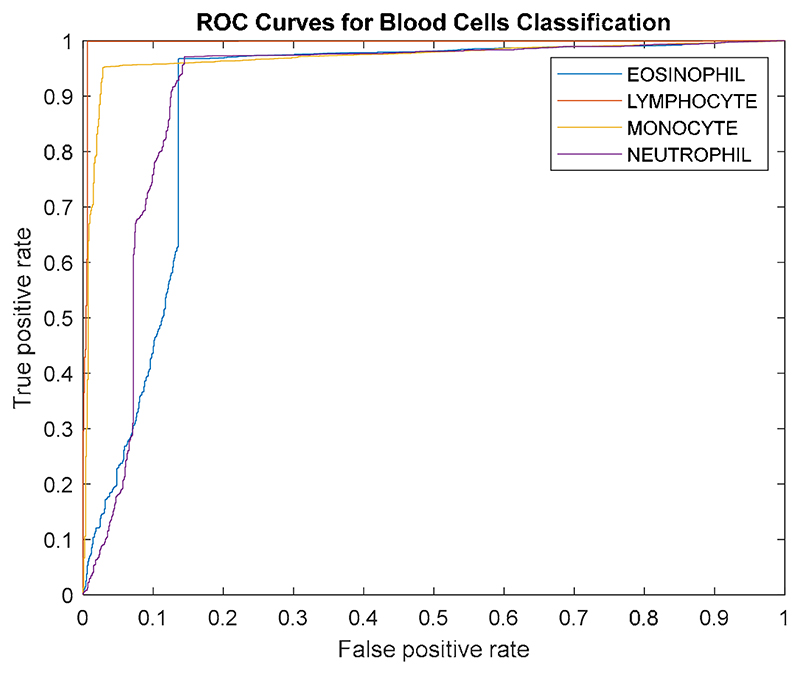
The ROC curve of the proposed model.

Five multi-classification measurements are implemented to evaluate the proposed DLBCNet. Considering the contingency, we carry out five runs. The classification performance of the proposed DLBCNet by five runs is presented in Table 4. The average accuracy, sensitivity, precision, specificity, and f1-score per class by five runs are given in Table 5. The average accuracy, average sensitivity, average precision, average specificity, and average f1-score of the proposed model are 95.05%, 93.25%, 97.75%, 93.72%, and 95.38%, accordingly. All the measurements per class of the proposed DLBCNet are greater than 90%. In particular, our model can achieve promising average accuracy for each class. The ROC curve is presented in Figure 7. The AUC values for eosinophil, lymphocyte, monocyte, and neutrophil are 0.8922, 0.9957, 0.9694, and 0.9091. Generally speaking, it can be concluded that our proposed model is an effective tool for the multi-classification of blood cells.

### 4.3 Comparison of Different Backbone Models

The pre-trained ResNet50 is selected as the backbone model for the proposed DLBCNet. There are numerous famous pre-trained CNN models, such as AlexNet, VGG, ResNet18, and MobileNet. The classification performance of different backbones is demonstrated in Table 6.

The proposed DLBCNet with the pre-trained ResNet50 as the backbone model can almost yield the best average accuracy, average sensitivity, average precision, average specificity, and average f1-score compared with other pre-trained models. The residual connection can improve the classification performance. More layers in ResNet50 can extract better features than ResNet18. Therefore, the pre-trained ResNet50 is utilized as the backbone of the proposed DLBCNet.

Using ResNet50 as the backbone model can obtain better results than other backbone models. The reason is that the residual connection in ResNet50 can improve the classification performance. Even though the residual connection is still in ResNet18, deeper layers can extract better features. In this situation, using ResNet50 as the backbone model has better performance than ResNet18.

### 4.4 Effects of the Proposed BCGAN

The proposed BCGAN is applied to generate synthetic images based on blood cell images to improve the classification performance. We create the mixed-BCGAN dataset based on these synthetic and original images. Meanwhile, the original GANs are compared with the proposed BCGAN to prove its superiority.

The comparison of the classification performance for the mixed and original datasets is demonstrated in Table 7. We test this comparison in five different backbone models to avoid fortuity. These models can yield better classification performance in the mixed-BCGAN dataset than in the mixed-GAN and original datasets. In conclusion, the proposed BCGAN is useful for diagnosing blood cells.

### 4.5 Effects of RNNs

Three RNNs are implemented as the classifier to replace the last five layers of the backbone model, which are ELM, RVFL, and SNN. The training time of RNNs can be less than traditional CNN models because of the simple structure and fixed randomized parameters. At the same time, RNNs can achieve promising results.

The effects of RNNs are given in Table 8. The classification results using the last five layers are not as good as those using three RNNs. It can be clearly concluded that the three RNNs used to substitute the last five layers can achieve better classification performance. The RNNs can have positive effects on blood cell classification.

### 4.6 Effects of ETRN

The performance of RNNs can vary with the randomized weights and biases. We propose the ETRN by combining three RNNs to improve classification performance. The effects of the proposed ETRN are shown in Table 9.

The average accuracy per class of ensemble network (DLBCNet) is generally the best, except for eosinophil. The accuracy of eosinophil is only 0.9% less than the best from ResNet50-RVFL. Therefore, the proposed ETRN can improve the multi-classification performance of blood cells.

### 4.7 Comparison with State-of-the-Art Methods

The proposed DLBCNet is compared to other state-of-the-art methods on the same public dataset, including CNN-AdaboostM1 [18] and the Xception-LSTM [20]. The comparison results of the proposed DLBCNet with other state-of-the-art methods are provided in Table 10.

Our model can yield the best average accuracy, average sensitivity, average precision, and average f1-score compared with other state-of-the-art methods. The Xception-LSTM achieved the best average specificity of 98.43%, which is 4.7% higher than our model. The comparison results suggest that the proposed DLBCNet is an accurate model for classifying blood cells.

## 5 Conclusions

The paper put forward a novel network for the classification of blood cells, which is called DLBCNet. We propose a new specifical model for blood cells (BCGAN) to generate synthetic images. The ResNet50 pre-trained on the ImageNet dataset is implemented as the backbone model, which is modified and fine-tuned based on blood cells. The modified ResNet50 is applied as the feature extractor. The extracted features are fed to the proposed ETRN, which combines three RNNs to improve the multi-classification performance of blood cells. The average accuracy, average sensitivity, average precision, average specificity, and average-f1-score of the proposed model are 95.05%, 93.25%, 97.75%, 93.72%, and 95.38%, accordingly.

In future research, we shall apply the proposed model to other public blood cell datasets to prove its generality. Additionally, other recent technology will be implemented in future research, such as MOCO, CLIP, and so on. Moreover, we will try to segment blood cell images.

## Figures and Tables

**Table 1:** Acronyms and full explanations.

Acronym	Full Explanation
DL	Deep learning
NLP	Natural language processing
CNNs	Convolutional neural networks
FC	Fully connected
BN	Batch normalization
GANs	Generative adversarial networks
RNNs	Randomized neural networks
ELM	Extreme learning machine
RVFL	Random vector functional link
SNN	Schmidt neural network
SVM	Support vector machine
Std	Standard deviation
ROC	Receiver operating characteristic
AUC	Area under the curve

**Table 2:** The comparison of the original dataset with the mixed dataset.

Dataset	Class	Training Set		Testing Set
		Original Images	Synthetic Images	Original Images
Original	Eosinophil	2184	0	936
	Lymphocyte	2172	0	931
	Monocyte	2169	0	929
	Neutrophil	2186	0	937

Mixed-GAN	Eosinophil	2184	3000	936
	Lymphocyte	2172	3000	931
	Monocyte	2169	3000	929
	Neutrophil	2186	3000	937

Mixed-BCGAN	Eosinophil	2184	3000	936
	Lymphocyte	2172	3000	931
	Monocyte	2169	3000	929
	Neutrophil	2186	3000	937

**Table 3:** The hyper-parameter setting of the proposed DLBCNet.

Hyper-Parameter	Value
Mini-batch size	10
Max-epoch	1
Initial learning rate	10^−4^
Z	400

**Table 4:** The performance of the proposed DLBCNet per class (%).

Run	Class	Accuracy	Sensitivity	Precision	Specificity	F1-Score
1	Eosinophil	94.31	91.00	95.41	86.54	93.15
	Lymphocyte	98.74	99.76	99.95	99.84	99.85
	Monocyte	95.77	87.66	99.97	99.91	93.41
	Neutrophil	93.90	93.79	95.41	87.19	94.59
2	Eosinophil	95.76	92.83	96.74	90.46	94.74
	Lymphocyte	97.63	100.00	100.00	100.00	100.00
	Monocyte	94.33	86.55	99.97	99.91	92.78
	Neutrophil	92.05	93.95	94.40	84.83	94.17
3	Eosinophil	96.26	92.28	97.58	92.72	94.86
	Lymphocyte	97.42	100.00	99.97	99.92	99.99
	Monocyte	94.41	87.66	99.97	99.91	93.41
	Neutrophil	91.68	94.51	93.95	83.89	94.23
4	Eosinophil	95.50	91.80	96.74	90.36	94.20
	Lymphocyte	97.53	99.60	100.00	100.00	99.81
	Monocyte	94.56	87.50	99.97	99.91	93.32
	Neutrophil	91.55	93.15	93.98	83.75	93.56
5	Eosinophil	95.68	92.52	96.74	90.42	94.58
	Lymphocyte	97.63	100.00	100.00	100.00	100.00
	Monocyte	94.35	86.54	100.00	100.00	92.79
	Neutrophil	91.96	93.95	94.27	84.53	94.11

**Table 5:** The average multi-classification measurements of the proposed DLBCNet by runs (%).

Class	Accuracy	Sensitivity	Precision	Specificity	F1-Score
Eosinophil	95.50	92.09	96.64	90.16	94.31
Lymphocyte	97.79	99.87	99.98	99.95	99.93
Monocyte	94.68	87.18	99.98	99.93	93.14
Neutrophil	92.23	93.87	94.40	84.84	94.13

**Table 6:** The classification performance of different backbones (%).

Backbone	Class	Accuracy	Sensitivity	Precision	Specificity	F1-Score
AlexNet	Eosinophil	76.22	45.51	86.45	36.49	45.49
	Lymphocyte	84.26	49.94	97.38	51.73	53.01
	Monocyte	80.82	37.41	96.97	46.53	43.72
	Neutrophil	59.06	75.51	55.20	37.20	38.36
ResNet18	Eosinophil	94.20	88.20	96.20	88.56	92.02
	Lymphocyte	97.34	99.47	99.85	99.57	99.66
	Monocyte	94.45	87.32	99.81	99.35	93.15
	Neutrophil	90.07	90.91	92.77	80.75	91.83
MobileNet	Eosinophil	94.77	89.65	96.47	89.44	92.93
	Lymphocyte	97.65	99.73	99.92	99.77	99.83
	Monocyte	94.75	88.30	99.78	99.27	93.69
	Neutrophil	90.90	91.37	93.50	82.42	92.42
VGG	Eosinophil	73.00	34.03	85.95	40.60	40.82
	Lymphocyte	73.34	59.62	75.23	40.93	52.27
	Monocyte	83.08	38.43	96.76	60.59	47.39
	Neutrophil	70.88	62.77	72.24	53.63	47.26
ResNet50	Eosinophil	95.50	92.09	96.64	90.16	94.31
	Lymphocyte	97.79	99.87	99.98	99.95	99.93
	Monocyte	94.68	87.18	99.98	99.93	93.14
	Neutrophil	92.23	93.87	94.40	84.84	94.13

**Table 7:** The comparison of the classification performance for the mixed and original datasets (%).

Backbone	Dataset	Class	Accuracy	Sensitivity	Precision	Specificity	F1-Score
AlexNet	Original	Eosinophil	53.71	41.45	57.81	24.74	48.28
		Lymphocyte	72.92	24.06	87.73	43.58	37.76
		Monocyte	52.80	37.89	58.10	23.05	45.87
		Neutrophil	73.72	5.02	97.25	37.90	9.54
	Mixed-GAN	Eosinophil	70.56	30.88	83.84	39.00	45.13
		Lymphocyte	74.78	56.93	81.24	54.92	66.95
		Monocyte	68.15	49.95	77.60	42.49	60.78
		Neutrophil	58.11	20.81	73.53	20.86	32.44
	Mixed-BCGAN	Eosinophil	76.22	45.51	86.45	36.49	45.49
		Lymphocyte	84.26	49.94	97.38	51.73	53.01
		Monocyte	80.82	37.41	96.97	46.53	43.72
		Neutrophil	59.06	75.51	55.20	37.20	38.36
ResNet18	Original	Eosinophil	90.51	76.40	95.23	84.25	84.78
		Lymphocyte	93.54	98.23	99.25	97.91	98.74
		Monocyte	87.88	74.68	99.14	96.66	85.19
		Neutrophil	81.05	87.50	85.24	66.50	86.36
	Mixed-GAN	Eosinophil	91.29	82.16	94.35	82.96	87.83
		Lymphocyte	98.06	100.00	99.96	99.89	99.98
		Monocyte	93.98	83.96	99.75	99.11	91.18
		Neutrophil	89.73	91.04	91.63	78.47	91.33
	Mixed-BCGAN	Eosinophil	94.20	88.20	96.20	88.56	92.02
		Lymphocyte	97.34	99.47	99.85	99.57	99.66
		Monocyte	94.45	87.32	99.81	99.35	93.15
		Neutrophil	90.07	90.91	92.77	80.75	91.83
MobileNet	Original	Eosinophil	92.47	82.48	95.82	86.84	88.65
		Lymphocyte	95.77	98.60	98.80	96.63	98.70
		Monocyte	92.73	83.53	99.68	98.85	90.89
		Neutrophil	87.48	89.75	90.41	75.83	90.08
	Mixed-GAN	Eosinophil	92.87	82.48	96.35	88.33	88.88
		Lymphocyte	96.85	99.79	99.70	99.15	99.74
		Monocyte	93.52	85.25	99.96	99.88	92.02
		Neutrophil	88.01	91.57	90.31	76.00	90.93
	Mixed-BCGAN	Eosinophil	94.77	89.65	96.47	89.44	92.93
		Lymphocyte	97.65	99.73	99.92	99.77	99.83
		Monocyte	94.75	88.30	99.78	99.27	93.69
		Neutrophil	90.90	91.37	93.50	82.42	92.42
VGG	Original	Eosinophil	65.68	19.66	81.09	25.81	31.65
		Lymphocyte	62.60	21.70	71.12	23.46	33.25
		Monocyte	74.85	0.00	100.00	0.00	0.00
		Neutrophil	43.59	53.15	40.59	23.07	46.03
	Mixed-GAN	Eosinophil	60.65	23.18	73.19	22.44	35.21
		Lymphocyte	74.34	1.93	97.87	28.57	3.79
		Monocyte	46.58	88.05	32.85	30.29	47.84
		Neutrophil	74.95	0.21	100.00	100.00	0.43
	Mixed-BCGAN	Eosinophil	73.00	34.03	85.95	40.60	40.82
		Lymphocyte	73.34	59.62	75.23	40.93	52.27
		Monocyte	83.08	38.43	96.76	60.59	47.39
		Neutrophil	70.88	62.77	72.24	53.63	47.26
ResNet50	Original	Eosinophil	92.86	86.11	95.39	87.51	90.52
		Lymphocyte	93.85	98.26	99.89	99.52	99.07
		Monocyte	87.21	73.63	99.56	98.42	84.65
		Neutrophil	82.68	91.25	86.43	71.61	88.77
	Mixed-GAN	Eosinophil	93.82	87.71	95.85	87.62	91.60
		Lymphocyte	96.39	98.28	99.78	99.35	99.02
		Monocyte	92.26	81.92	99.54	98.32	89.87
		Neutrophil	89.12	92.64	91.67	78.84	92.15
	Mixed-BCGAN	Eosinophil	95.50	92.09	96.64	90.16	94.31
		Lymphocyte	97.79	99.87	99.98	99.95	99.93
		Monocyte	94.68	87.18	99.98	99.93	93.14
		Neutrophil	92.23	93.87	94.40	84.84	94.13

**Table 8:** The effects of RNNs (%).

Model	Class	Accuracy	Sensitivity	Precision	Specificity	F1-Score
ResNet50	Eosinophil	92.38	88.54	93.66	82.31	91.0
	Lymphocyte	97.01	98.49	99.81	99.43	99.14
	Monocyte	93.13	82.80	99.73	99.05	90.48
	Neutrophil	88.41	86.23	92.14	78.54	89.09
ResNet50-ELM	Eosinophil	94.30	91.56	95.22	86.47	93.35
	Lymphocyte	97.49	99.92	99.84	99.52	99.88
	Monocyte	93.39	83.20	99.81	99.33	90.75
	Neutrophil	91.35	92.09	94.06	83.74	93.07
ResNet50-RVFL	Eosinophil	96.42	92.99	97.56	92.70	95.22
	Lymphocyte	95.67	99.92	99.84	99.52	99.88
	Monocyte	91.11	80.73	99.89	99.61	89.30
	Neutrophil	88.62	94.11	91.96	79.60	93.02
ResNet50-SNN	Eosinophil	94.96	90.29	96.52	89.64	93.30
	Lymphocyte	97.16	99.92	99.95	99.84	99.93
	Monocyte	93.32	84.71	99.76	99.16	91.62
	Neutrophil	90.46	92.99	93.07	81.74	93.03

**Table 9:** The effects of ETRN (%).

Model	Class	Accuracy	Sensitivity	Precision	Specificity	F1-Score
ResNet50-ELM	Eosinophil	94.30	91.56	95.22	86.47	93.35
	Lymphocyte	97.49	99.92	99.84	99.52	99.88
	Monocyte	93.39	83.20	99.81	99.33	90.75
	Neutrophil	91.35	92.09	94.06	83.74	93.07
ResNet50-RVFL	Eosinophil	96.42	92.99	97.56	92.70	95.22
	Lymphocyte	95.67	99.92	99.84	99.52	99.88
	Monocyte	91.11	80.73	99.89	99.61	89.30
	Neutrophil	88.62	94.11	91.96	79.60	93.02
ResNet50-SNN	Eosinophil	94.96	90.29	96.52	89.64	93.30
	Lymphocyte	97.16	99.92	99.95	99.84	99.93
	Monocyte	93.32	84.71	99.76	99.16	91.62
	Neutrophil	90.46	92.99	93.07	81.74	93.03
DLBCNet	Eosinophil	95.50	92.09	96.64	90.16	94.31
	Lymphocyte	97.79	99.87	99.98	99.95	99.93
	Monocyte	94.68	87.18	99.98	99.93	93.14
	Neutrophil	92.23	93.87	94.40	84.84	94.13

**Table 10:** Comparison with other state-of-the-art methods (%).

Method	Average-Accuracy	Average-Sensitivity	Average-Precision	Average-Specificity	Average-F1-Score
CNN-AdaboostM1	88.00	85.90	-	-	-
Xception-LSTM	90.79	-	95.83	98.43	95.00
DLBCNet	95.05	93.25	97.75	93.72	95.38

## Data Availability

The dataset can be downloaded at https://www.kaggle.com/datasets/paultimothymooney/blood-cells (accessed on 2 January 2023).
